# Influence of air mapping errors on the dosimetric accuracy of prostate CBCT‐guided online adaptive radiation therapy

**DOI:** 10.1002/acm2.14057

**Published:** 2023-06-05

**Authors:** Olga M. Dona Lemus, Sean Tanny, Michael Cummings, Matthew Webster, Joshua Wancura, Hyunuk Jung, Yuwei Zhou, Jihyung Yoon, Matthew Pacella, Dandan Zheng

**Affiliations:** ^1^ Department of Radiation Oncology University of Rochester Medical Center New York New York USA

**Keywords:** adaptive therapy, air mapping errors, prostate cancer, synthetic CT

## Abstract

**Purpose:**

CBCT‐guided online adaptive radiotherapy (oART) plans presently utilize daily synthetic CTs (sCT) that are automatically generated using deformable registration algorithms. These algorithms may have poor performance at reproducing variable volumes of gas present during treatment. Therefore, we have analyzed the air mapping error between the daily CBCTs and the corresponding sCT and explored its dosimetric effect on oART plan calculation.

**Methods:**

Abdominopelvic air volume was contoured on both the daily CBCT images and the corresponding synthetic images for 207 online adaptive pelvic treatments. Air mapping errors were tracked over all fractions. For two case studies representing worst case scenarios, dosimetric effects of air mapping errors were corrected in the sCT images using the daily CBCT air contours, then recalculating dose. Dose volume histogram statistics and 3D gamma passing rates were used to compare the original and air‐corrected sCT‐based dose calculations.

**Results:**

All analyzed patients showed observable air pocket contour differences between the sCT and the CBCT images. The largest air volume difference observed in daily CBCT images for a given patient was 276.3 cc, a difference of more than 386% compared to the sCT. For the two case studies, the largest observed change in DVH metrics was a 2.6% reduction in minimum PTV dose, with all other metrics varying by less than 1.5%. 3D gamma passing rates using 1%/1 mm criteria were above 90% when comparing the uncorrected and corrected dose distributions.

**Conclusion:**

Current CBCT‐based oART workflow can lead to inaccuracies in the mapping of abdominopelvic air pockets from daily CBCT to the sCT images used for the optimization and calculation of the adaptive plan. Despite the large observed mapping errors, the dosimetric effects of such differences on the accuracy of the adapted plan dose calculation are unlikely to cause differences greater than 3% for prostate treatments.

## 1 INTRODUCTION

In the modern era of radiation therapy, the need for patient setup reproducibility is critical. Reproducibility is affected by patient setup errors and internal organ motion resulting from normal physiological processes such as breathing, peristalsis, and cavity filling. Techniques for improving patient setup reproducibility include immobilization devices, image‐guided radiation therapy (IGRT), and planning structures such as internal target volumes (ITV) and planning organ at risk volumes (PRV).^1^ However, adding expansion margins to the clinical target volume to account for setup errors and internal organ motion also increases dose to healthy tissues. To best spare healthy tissue while maintaining target coverage, one option is to adapt treatments to the continuously changing patient anatomy.

Common strategies for adaptive radiotherapy (ART) have focused on treatment re‐planning to adjust for changes in the target volume due to weight loss and tumor response or changes in the clinical goals.^2^, ^3^, ^4^, ^5^, ^6^, ^7^, ^8^ Such changes were commonly detected through treatment image guidance systems or external diagnostic PET and MRI imaging.^9^, ^10^, ^11^, ^12^ Despite the mounting evidence of daily adaptation benefits such as improved local regional control and reduced toxicity, technical challenges around ART made it impractical to implement daily.^13^, ^14^, ^15^ Adaptive techniques have commonly been applied offline and reserved for selected cases where just a few adaptations were required during the treatment course.

Recent advances in applying artificial intelligence (AI) to auto‐segmentation, inverse planning, and faster dose calculation engines have enabled the possibility of more sophisticated online adaptive therapy (oART) approaches.^16^ Among other alternatives, the Varian Ethos system (Varian Medical Systems, Palo Alto, California, USA) offers a solution capable of automated daily adaptive treatment with minimal clinical resources required as it circumvents the need for re‐simulation or dosimetrist‐generated re‐planning. An AI‐driven auto‐segmentation tool then defines site‐specific influencer structures that are used to drive a structure‐guided deformation for the relevant target volumes as defined in the original plan. These influencer structures and resulting target volumes can be manually edited by the clinical team which presents a trade‐off between accuracy and time efficiency. The adaptive plan optimization and dose are calculated on the sCT image using the final adapted structure set.

Radiation dose calculations are highly sensitive to the heterogeneity of the media where the energy fluence is being delivered and rely on the accuracy of the acquired images to determine the mass and electron density of such media.^17^ Thus, errors in the synthetic image due to incorrectly mapped air volumes may eventually lead to errors in the dose distribution. B‐spline DIR algorithms are generally accurate around small deformations. Yet, some limitations persist around large deformations or the addition/removal of new volumes such as air pockets.^18^ The abdominopelvic area is particularly known for its air volume changes due to rectal and bowel filling changes and B‐spline DIR methods are currently unable to produce synthetic images capable of accurately deforming or generating the true volume of air contained in the abdominal cavity. Since clinical decisions are made based on the dose distribution calculated on a synthetic image during adaptive treatment, the effect of volume mapping errors cannot be ignored.

The primary objectives of this study are to assess the accuracy of air volume mapping between CBCT and sCT images and to evaluate the dosimetric impact of air mapping inaccuracies in the prostate oART workflow. This evaluation is necessary and important to determine whether air mapping inaccuracies give rise to concerns in utilizing the current oART workflow and whether further corrections are needed.

## 2 MATERIALS AND METHODS

### 2.1 Study dataset

Daily CBCT and respective synthetic CT images were extracted from 207 daily adaptive sessions corresponding to six prostate cancer patients (P1 – P6) treated on a Varian Ethos (Varian Medical Systems, Palo Alto, California, USA) from July 2021 to July 2022 (IRB 00007059).

Planning CT images were acquired on a LightSpeed‐RT16 (GE Healthcare), helical mode using a prostate protocol at 120 kVp with a matrix size of 512×512 pixels, 0.13 cm axial resolution and 0.25 cm slice thickness. The CBCT images were acquired on the Ethos integrated imager (Varian Medical Systems, Palo Alto, California, USA) using the pelvis CBCT mode with a matrix size of 512×512 pixels, slice thickness 0.199 cm at 125 kVp. The sCT images were automatically created on Varian Ethos using structure‐guided DIR between the planning CT and the daily CBCT. The influencer structures used to guide the deformation were bladder, prostate (in the intact‐prostate treatment cases) and rectum volumes. These influencer structures were generated using an AI‐based proprietary algorithm from Varian and corrected by the radiation oncologist for each adaptive session.

The six adaptive cases included three prostate bed and lymph (LN) nodes cases, one prostate bed case; one prostate, seminal vesicles (SV) and LN case; and one prostate and LN nodes case. For these pelvic adaptive treatments, 9 or 12‐field static IMRT fields were used to reduce on‐couch calculation time. The abdominopelvic air was not overridden during treatment planning, consistent with our standard clinical practice. All patients were given standard full‐bladder, empty rectum instructions to follow for simulation and during adaptive treatments. Patient‐specific prescription regimens and treatment techniques are summarized in Table 1.

**TABLE 1:** Summary of the patients and treatment characteristics of the studied cohort.

	Site	Prescription	# Fx	Treatment	anti‐gas medication
P1	Prostate Bed and LN	Phase 1 (45 Gy/ 25 Fx) Phase 2 (23.4 Gy/ 13 Fx to prostate bed)	38	12 Fields Static IMRT	Started FX16
P2	Prostate, SV and LN	Phase 1 (62.5 Gy to prostate; 50 Gy to SV and; 45 Gy to LN/25 Fx) Phase 2 (7.5 Gy/3 Fx to prostate)	28	12 Fields Static IMRT	Started FX8 due to persistent gas
P3	Prostate, SV and LN	Phase 1 (54 Gy to prostate and SV; 59.4 Gy to node positive and 48.6 Gy to LN /27 Fx) Phase 2 (24 Gy/12 Fx to prostate)	39	12 Fields Static IMRT for phase 1 and 9 Fields Static IMRT for phase 2	None
P4	Prostate Bed and LN	Phase 1 (45 Gy/25 Fx) Phase 2 (23.4 Gy/13 Fx to prostate bed)	38	12 Fields Static IMRT	None
P5	Prostate Bed	Phase 1 (45 Gy/25 Fx) Phase 2 (23.4 Gy/13 Fx to prostate bed)	38^a^	9 Fields Static IMRT	None
P6	Prostate Bed and LN	Phase 1 (45 Gy/25 Fx) Phase 2 (23.40 Gy/ 3 Fx to prostate bed)	38	12 Fields Static IMRT for phase 1 and 9 Fields Static IMRT for phase 2	None

^a^ At the time of data collection, only 26 fractions had been delivered.

### 2.2 Abdominopelvic air volume assessment

The abdominopelvic air volume was segmented on the CBCT and sCT images by applying a threshold of −1000 to −300 HU inside the body contour on both images. The air volume analysis was restricted to the size of the CBCT image as the CBCT image has shorter longitudinal range than the planning CT.

Air volume was tracked for both images for each patient for the duration of the treatment. Statistical metrics on volume and volume differences were used to assess variability of abdominal air and air mapping accuracy between the CBCT and the sCT images, including maximum, minimum, interquartile range (IQR), median, mean, distribution probability, and outliers. The mapping error is defined as shown in Equation (1).

(1) $$Mapping\;Error\;\;=\;\;\left(V_{Air}^{CBCT}-V_{Air}^{sCT}\right)$$

### 2.3 Dosimetric assessment

**FIGURE 1:**
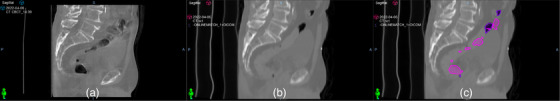
(a) CBCT image. (b) Registered sCT image. (c) Corrected sCT. The sCT air volume (purple) would be overridden with HU = 0 and the CBCT air (magenta) would be overridden with HU = −700 in the corrected sCT. Sagittal images from P3‐FX21.

For the worst‐case scenario, defined as the largest air volume mapping error found between the synthetic and CBCT images, the dosimetric effect was assessed by recalculating the plan on a corrected synthetic image. The corrected sCT was created by overriding the abdomino‐pelvic air in the synthetic image with water density (0 HU) while a structure corresponding to the CBCT air segmentation was added with an assigned density of −700 HU (the average density for abdominal air was measured at – 695.7 HU). Air volume correction was limited to the overlapping volume between the CBCT image and the sCT image. The CBCT image size is generally smaller than the synthetic image due to the longitudinal extent of the Ethos kV‐CBCT imager. Figure 1 demonstrates the process of creating a corrected sCT.

The corrected dose matrix was recalculated using the session‐generated adaptive plan in Eclipse v15.6 (Varian Medical Systems, Palo Alto, California, USA) using Acuros v15.6.06 (Varian Medical Systems, Palo Alto, California, USA) with a resolution of 0.25 cm and dose to medium with heterogeneity correction). The uncorrected dose matrix was also recalculated in Eclipse to eliminate any confounding dose difference created by the differences in beam data and dose calculation algorithm between Ethos and Eclipse. Dosimetric differences were reported as per Equation (2).

(2) $$\begin{array}{c} Dose\;Difference\;\left[\%\right]=100\times \frac{\left(D_{uncorrected}-D_{corrected}\right)}{D_{corrected}} \end{array}$$

The metrics used to quantify the dosimetric effect of air volume mapping errors were the 3D gamma passing rate^19^ using 1%, 2%, and 3% dose difference, and 1 , 2 and 3 mm distance to agreement relative to the maximum dose (global) and DVH‐based metrics for the PTV such as Max Dose, Mean Dose, Min Dose, D98, and D95. The gamma passing rates were calculated for the entire dose volume and for PTV specific volumes. The 3D gamma index was calculated in SlicerRT^20^ using the gamma plastimatch algorithm. This algorithm defines that two images are similar at a given location if there is a voxel with similar intensity nearby in the comparison image. The gamma passing rate was defined as a percentage of points satisfying the dose difference and distance to agreement conditions previously described.

## 3 RESULTS

### 3.1 Abdominopelvic air volume assessment

**FIGURE 2:**
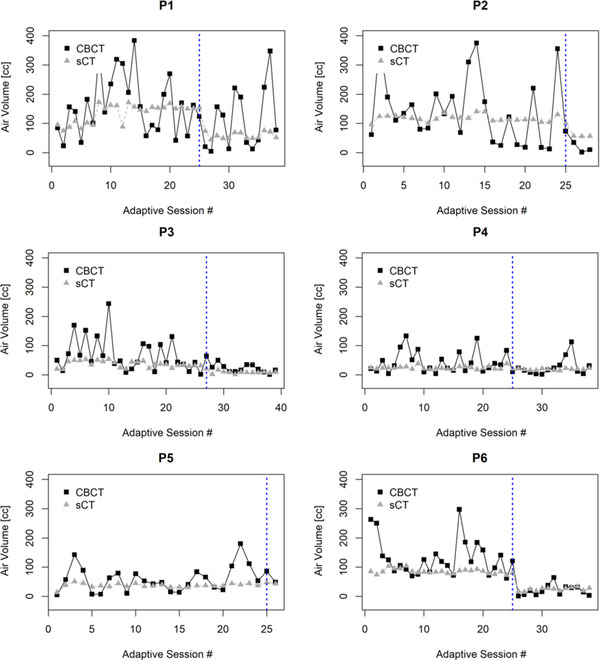
Air volume tracking during treatment of the studied patients P1‐P6. Dark Grey plots show the air volume in the daily CBCT image while light grey plots show the air volume of the daily sCT image. The dotted blue line indicates the ending of phase 1.

Daily air volume variations were tracked on the daily CBCT image and corresponding sCT for 6 oART patients as shown in Figure 2.

As shown in Figure 2, CBCT images captured daily volume variations in abdominopelvic air cavities for all the patients, while the sCT failed to reproduce such variations. Among different patients, those that started with more gas on the simulation day also showed more daily variations (P1 −109.1 cc, P2 −110.4 cc, and P6 −64.3 cc), and those that started with less gas showed relatively less variations (P3 −27.2 cc, P4 −21.9 cc, and P5 −37.5 cc) though the variations were still much larger than captured by sCTs. It is worth noting that the drop in synthetic air volume for patients P1, P2, and P6 correspond to cone down boost treatments where the CBCT field of view was limited to the area surrounding the boosted volume. For these patients, there was a significant amount of air in the bowel for phase 1 that was not included in the cone down CBCT field of view for phase 2. Therefore, as can be seen in Figure 2, the sCT image and CBCT might show reduced total abdominopelvic air volume for the cone down phase of the treatment.

**FIGURE 3:**
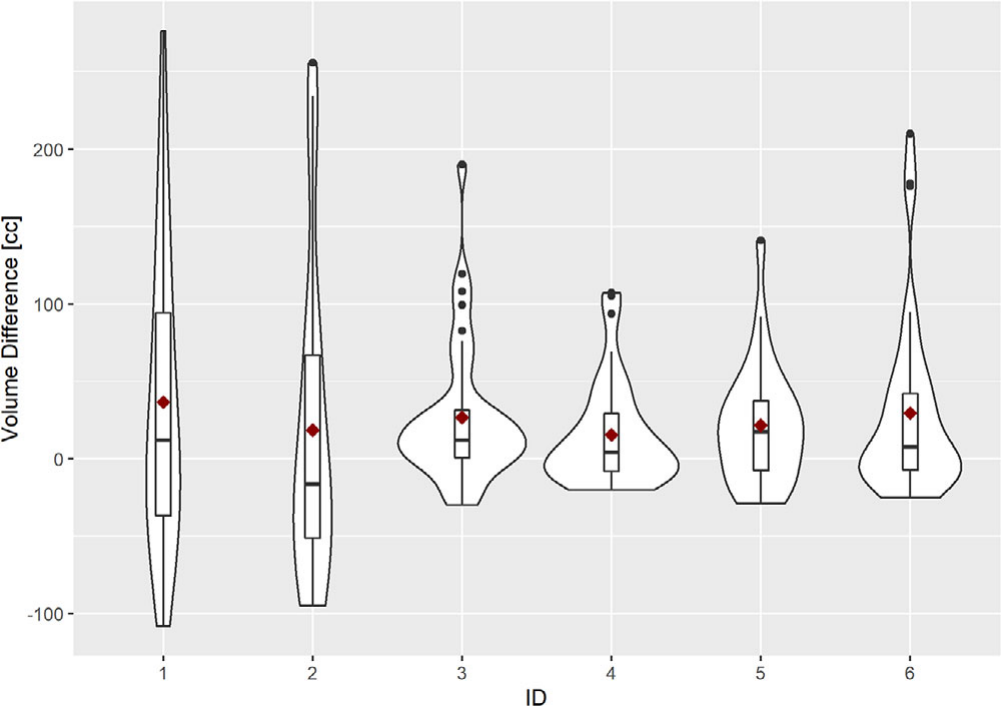
Air volume differences [cc] (air mapping error) between the daily CBCT and respective sCT for each patient (P1 ‐ P6). In the plots, the black horizontal line inside the box plot represents the median and the red dot represents the mean. The box in the center is the interquartile range (−25% to 25%), the black vertical line depicts the rest of the distribution, and the black dots represent the outliers. The shape of the violin illustrates the distribution probability.

The distribution of air‐mapping errors for each patient is shown in Figure 3. For patients P3, P4, P5, and P6, the mapping error is highly concentrated around the median. In contrast, in patients P1 and P2, the air mapping error varies throughout a wider range, which can be appreciated in the IQR. On average, P1 showed the highest mean air mapping error at 36.6 cc, while P4 showed the lowest mean air volume error at 15.5 cc. The largest air volume error among all the patients was found for P1, session 37 (P1‐FX37) at 276.3 cc and the largest percentage error intercepting a PTV was found for P3 session 21 (P3‐FX21) at 484.4% (108.2 cc). Additional metrics to evaluate the air mapping errors are shown in Table 2.

**TABLE 2:** Abdominal air mapping errors between the CBCT and the corresponding sCT.

	Mean Volume error (cc)	Median Volume error (cc)	Min error (cc)	Max error (cc)	IQR (cc)
P1	36.6	11.8	−108.1	276.1	131.0
P2	18.5	−16.4	−94.9	255.8	118.1
P3	26.8	11.7	−29.8	190.0	30.7
P4	15.5	4.2	−20.1	107.3	37.7
P5	21.7	17.4	−29.1	141.1	45.1
P6	29.4	7.5	−25.1	209.8	49.5

### 3.2 Dosimetric assessment

**FIGURE 4:**
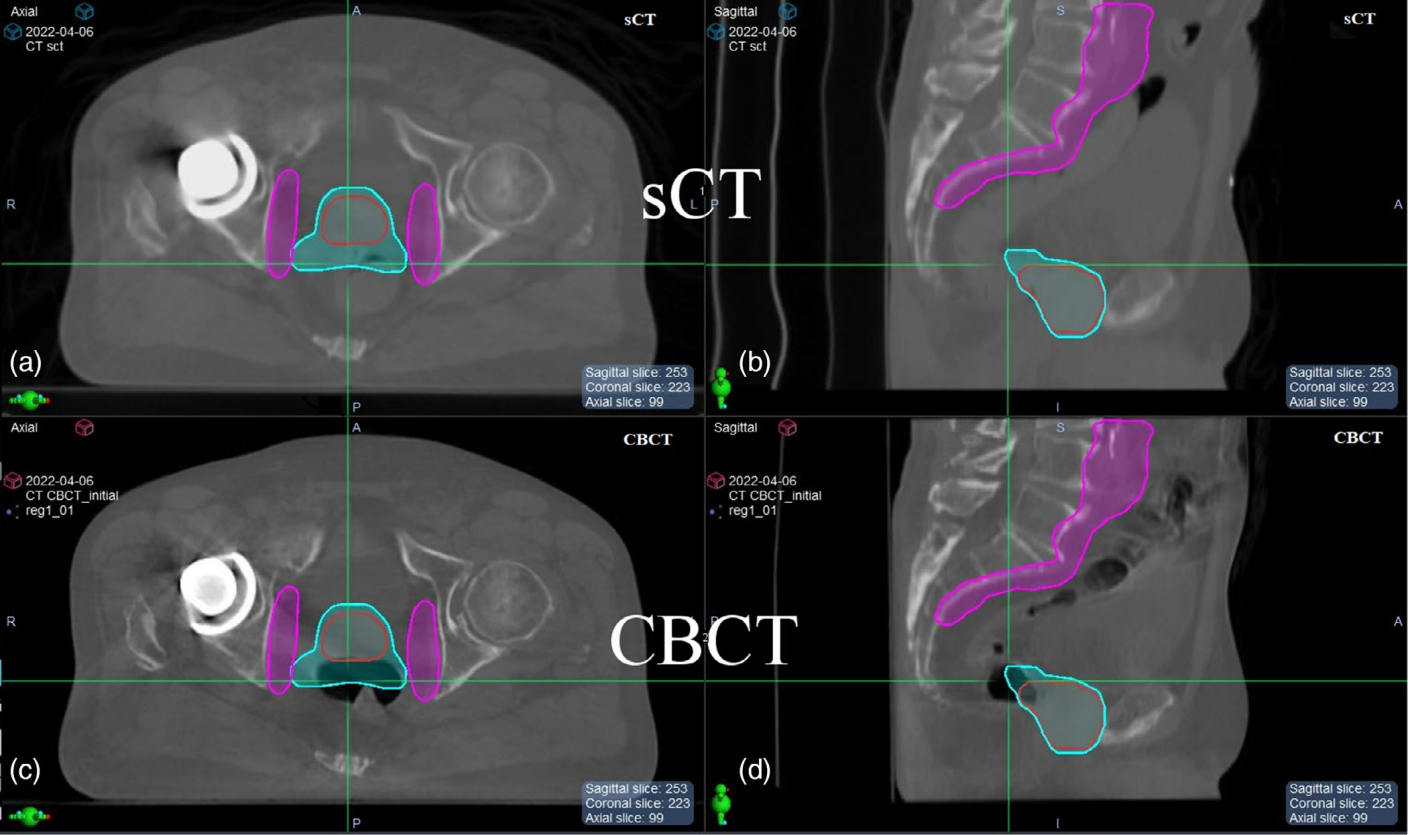
CBCT and respective sCT images for P3‐FX21 showing the differences in abdominopelvic air cavities. (a) sCT axial. (b) sCT sagittal. (c) CBCT axial. (d) CBCT sagittal. PTV4860 (magenta), PTV5400 (cyan), and PTV7800 (red) were overlaid on both images to highlight the proximity and overlap with the uncorrected air volume.

The dosimetric effect of uncorrected air volume was assessed for two cases. The first case, P3, session 21 (P3‐FX21), was chosen because it represented a worst‐case scenario. The planning CT contained a small volume of air (27.2 cc), while the daily CBCT had a large air pocket (130.5 cc) directly overlapping the PTV. The air volume overlap between the CBCT and the planning CT was 2.3 cc. Figure 4 shows the axial and sagittal views of sCT versus CBCT for P3‐FX21 with overlaid PTVs to illustrate proximity and overlap with air cavities. In particular, the rectal air for this fraction overlaps with the PTV prescribed 78 Gy [PTV7800 (red)] and with the PTV prescribed 54 Gy [PTV5400 (cyan)].

**FIGURE 5:**
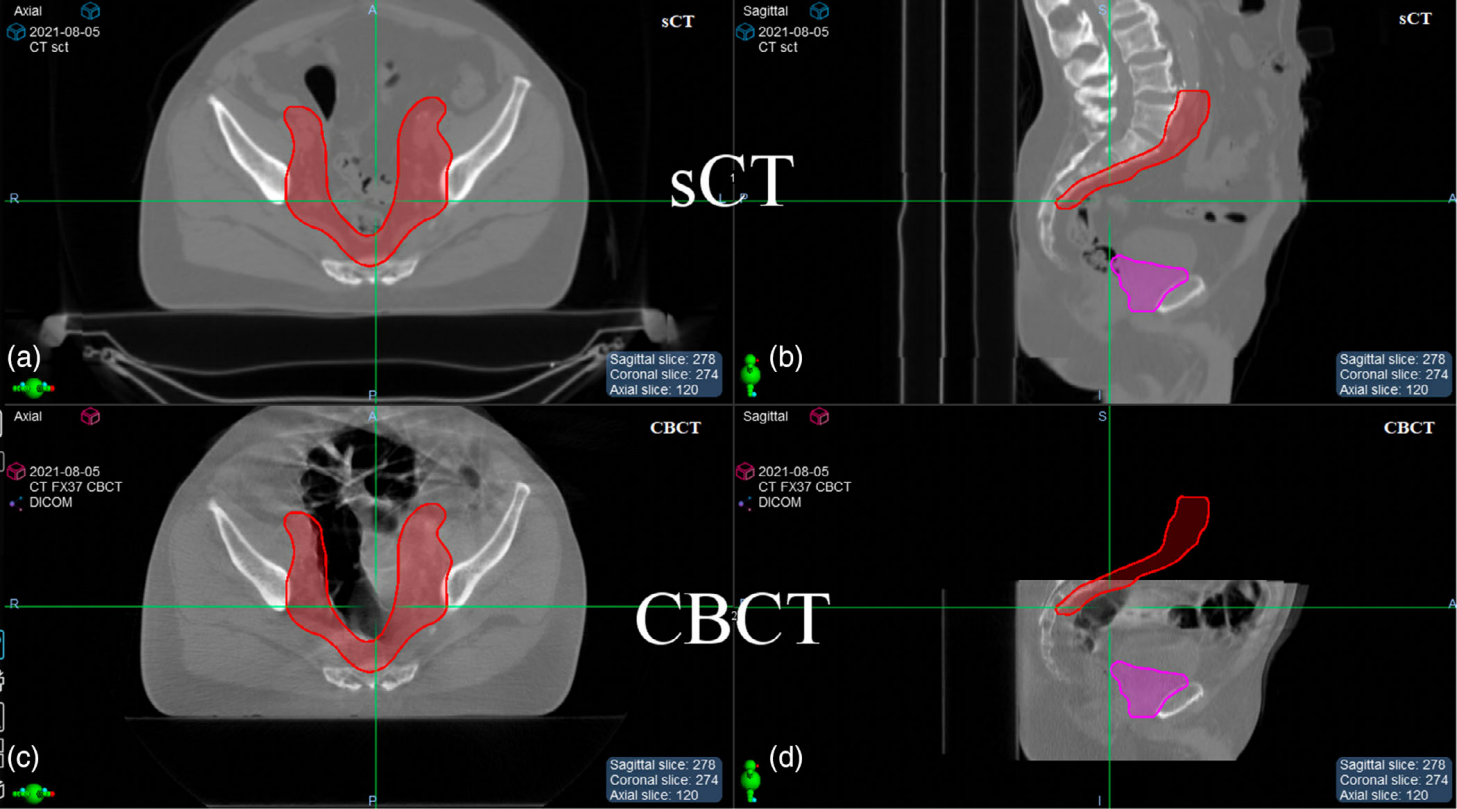
CBCT and respective sCT images for P1‐FX37 showing the differences in abdominopelvic air cavities. (a) sCT axial. (b) sCT sagittal. (c) CBCT axial. (d) CBCT sagittal. PTV6840 (magenta) and PTV4500 (red) were overlaid on both images to show the proximity and overlap with the uncorrected air volume.

The second case, P1, session 37 (P1‐FX37) was chosen because it had the largest absolute air mapping error in our dataset. Similarly, Figure 5 shows the axial and sagittal views of sCT vs CBCT for P1‐FX37. In this case, most of the air present in the CBCT image is contained in the bowel and does not overlap with the target treated for the boost session, PTV6840 (magenta). Because the air cavities are adjacent to PTV4500 (red) which was treated in the initial phase, to simulate the effect of the largest air volume mapping error, we instead calculated the initial plan for phase 1 for this case. It is worth pointing out that, due to the limited longitudinal range of CBCT for this phase 2 fraction, the superior portion of the abdomen is missing, which could likely contain additional air mapping errors adjacent to PTV4500. For P1‐FX37, the planning CT contained a small volume of air (75.3 cc), while the daily CBCT had a large air pocket (223.6 cc). The air volume overlap between the CBCT and the planning CT was 41.6 cc.

Dose statistics and gamma passing rates for uncorrected and corrected air volume are shown in Table 3. For P3‐FX21, we calculated a dose difference above 1% for D98 and D95 on PTV5400 and Min Dose and D98 for PTV7800. Despite showing an overall 3D gamma passing rate of 98.89% using the 1%/1 mm criteria, PTV specific gamma passing rates for PTV5400 and PTV7800 were lower than 90%. Gamma passing rates above 95% were calculated using 2%/2 mm and 3%/3 mm criteria. These results were consistent with the relative position of the air volume difference and the PTV location.

**TABLE 3:** Results for uncorrected and corrected air volume dose calculation and gamma passing rates (GPR).

uncorrected|corrected	Min dose (cGy)	D98 (cGy)	D95 (cGy)	Mean dose (cGy)	Max Dose (cGy)	GPR 1%/1 mm (%)	GPR 2%/2 mm (%)	GPR 3%/3 mm (%)
P3‐FX21						**98.89**	**99.85**	**99.99**
PTV4860 (180cgy/fx)	167|**167** (−0.04%)	181|**181** (0.00%)	184|**184** (0.00%)	188|**187** (0.34%)	225|**226** (−0.08%)	91.64	98.53	99.92
PTV5400 (200cGy/fx)	195|**195** (0.29%)	203|**205** (1.07%)	204|**202** (1.15%)	207|**206** (0.61%)	216|**215** (0.62%)	75.39	96.20	99.25
PTV7800 (200cGy/fx)	202|**197** (2.63%)	204|**202** (1.45%)	204|**206** (−0.65%)	207|**206** (0.56%)	214|**215** (−0.05%)	75.49	96.07	99.10
P1‐FX37						**92.86**	**97.49**	**99.24**
PTV4500 (180cGy/fx)	115|**114** (0.95%)	175|**178** (1.27%)	179|**180** (0.70%)	184|**185** (1.01%)	195|**199** (1.44%)	63.73	78.60	92.03
PTV6840 (180cGy/fx)	107**|107** (0.25%)	151|**151** (0.00%)	168|**168** (0.00%)	185|**185** (0.12%)	202|**203** (−0.51%)	96.61	99.91	100

In the case of the simulated scenario for P1‐FX37, we calculated a dose difference greater than 1% for Max Dose, Mean Dose, and D98 on PTV4500. The specific passing rate for PTV4500 was 63.73% for 1%/1 mm and 78.60% for 2%/2 mm . The overall passing rate was above 90%.

**FIGURE 6:**
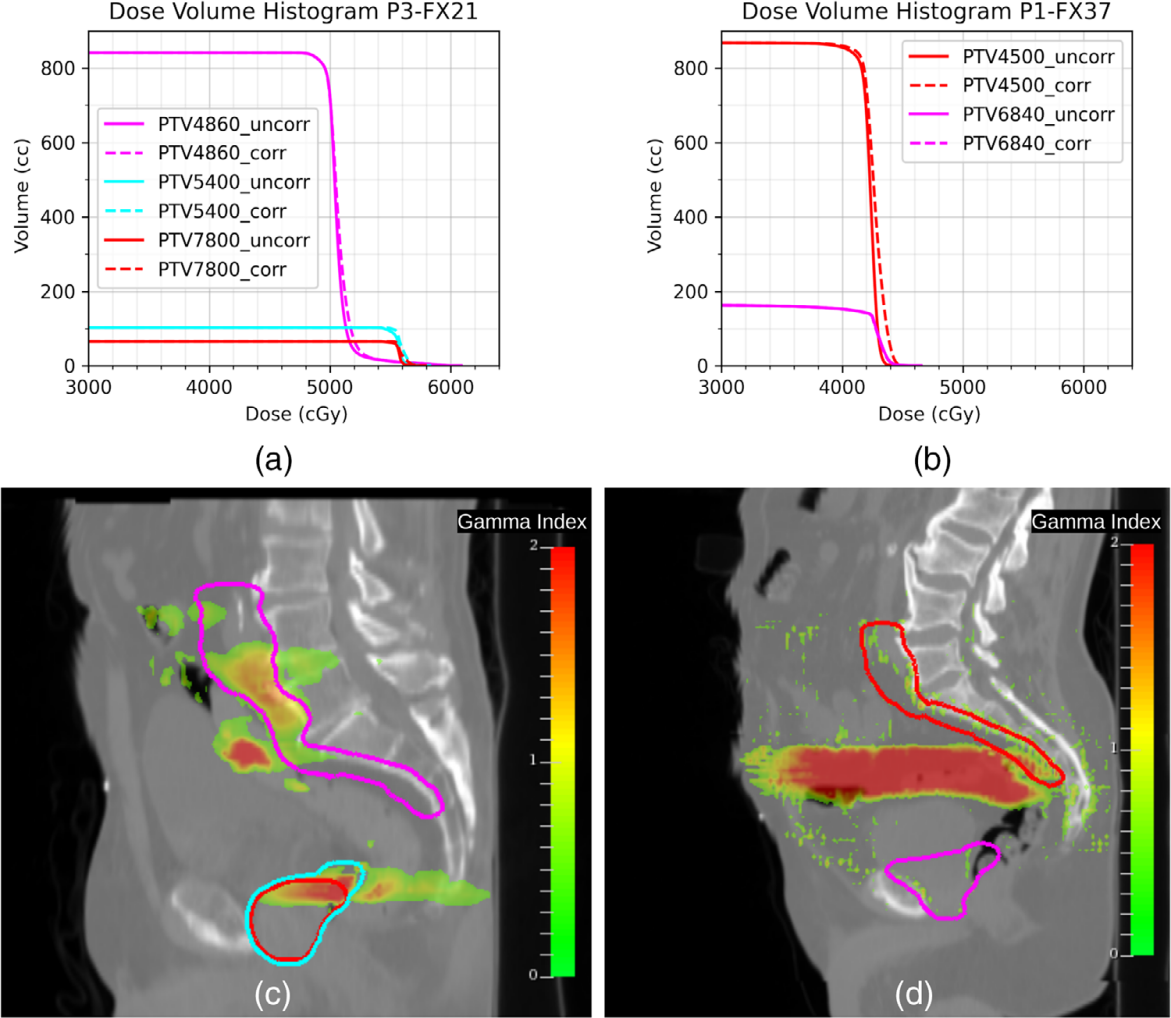
DVH and gamma overlay between uncorrected and corrected air volume calculation for a single fraction rescaled to full treatment dose. (a) DVH differences for PTV4860 (magenta), PTV5400 (cyan), and PTV7800 (red) for P3‐FX21. (b) DVH differences for PTV6840 (magenta) and PTV4500 (red) for P1‐FX37. (c) and (d) show respective gamma index overlay on the sCT. Gamma upper bound was set at 2 to simplify the analysis and a threshold was applied to the plot to show gamma 0.5.

To understand the dosimetric implications of uncorrected air mapping errors, Figure 6 shows the PTV DVHs for P3‐FX21 and P1‐FX37 and a gamma index overlay on the respective sCT image. Here, we visualized where the dose differences were greater and the correlation of such differences with the air volume mapping errors as shown in Figures 4 and 5. As expected for P3‐FX21, the gamma overlay shows areas where gamma is greater than one intercepting the PTV4860 (magenta), PTV5400 (cyan), and PTV7800 (red). For P3‐FX21, the areas of concern are mostly affecting PTV4500 (red).

## 4 DISCUSSION

In this study, we tracked the changes in abdominopelvic air volume over 207 sessions of pelvic oART treatments on the daily CBCT and corresponding sCT for treating primary and nodal targets of patients with either intact prostate or post‐prostatectomy prostate bed. Volume and location mapping errors of these air pockets directly affect the HU and electron density maps; hence, we also explored the dosimetric effect of the mapping errors in the dose distribution and its relevance.

The main advantage of oART is reducing treatment uncertainty by considering and adapting the treatment to daily variations of patients' anatomy. Yet for CBCT‐guided oART, the sCT used to optimize and calculate the adaptive treatment does not accurately account for air volume variability between daily and planning anatomy in the DIR between the two. However, despite recording large air volume mapping errors between the daily CBCT and corresponding sCT, delivered dose differences to the target volumes for evaluated metrics remained below 2.7%.

Several factors can affect the variability and volume of intestinal gas in patients such as dietary choices, digestive problems, intestinal infections, medications, and behavioral factors.^19^ In the cohort studied, all patients were recommended to follow a low fiber diet. However, for many patients, these draconian lifestyle changes can be challenging and may not be sufficient to control intestinal gas. For two patients who showed large daily gas volume variability (P1 and P2), the anti‐gas medication simethicone (Gas‐X) was prescribed mid‐course, although the effect was not apparent. This is consistent with reports of previous studies.^21^ The sCT image is created using DIR with the initial planning CT as the base, and the DIR algorithm cannot effectively handle air cavities due to large magnitudes of these deformations and the lack of contrast in air. Our study demonstrates that the DIR algorithm was unable to replicate the interfractional air volume variability of patients undergoing a full course of pelvic irradiation. Considerable air mapping errors were observed between the sCT and the CBCT images. The largest volume error observed was 276.3 cc.

Nearby anatomy changes such as daily bladder and rectal filling fluctuations play a role in the bowel gas position and volume. Despite the use of a bladder and rectal filling protocol, high volume changes were recorded during treatment. This observation is consistent with previous studies that have reported on the efficacy of different protocols in achieving consistent organ filling during treatment.^22^, ^23^ Furthermore, prolonged radiation therapy is known to generate proctitis and irritative biologic effects associated with increased gas production. It has been reported in the literature that 30–70% of patients experienced excessive flatulence after cancer treatment.^24^, ^25^, ^26^ This will likely produce a positive skew in the air mapping error. In this study, the distribution of air volume differences between CBCT and sCT were positively skewed indicating that the synthetic image generally underestimated the volume of air present in the image and that an increase of abdominopelvic air volume compared to the initial planning CT was observed. As the sCT more closely represents the air volume on the planning CT than on the daily CBCT, when more air is present in the CBCT compared to the planning CT, the sCT underestimates the volume of intestinal air.

The dosimetric effect of uncorrected air mapping errors was assessed for two case studies. The first was for a patient with little to no air in the planning CT, but a large air pocket in the PTV on the daily CBCT. The second was for the session with the largest absolute volume difference (276.3 cc) observed in our dataset. In both cases, the volume of uncorrected air was in close proximity to the targets. The largest target dose difference calculated using DVH‐based metric was 2.64%. All other target dose metrics reported dose differences below 1%. While gamma passing rates using 1%/1 mm criteria were above 90% when comparing uncorrected and corrected dose distributions for these two cases, previous studies by Zhang et al.^27^ and Han et al.^28^ have proposed that PTV‐specific analysis is more sensitive to MLC errors, and this has a higher correlation with DVH metrics. PTV‐specific gamma passing rates for the two case studies fell below 90% for a stringent 1%/1 mm criteria. However, the differences in PTV coverage as measured by D98 and D95 were still below 2%, reinforcing the idea that reduced passing rate caused by the air mapping error does not automatically imply a large change in delivered dose. Although not studied in this paper, nearby organs at risk could also be affected by air mapping errors. For the two studied cases, OAR‐specific gamma passing rates were above 95% except for the bowel space in P1‐FX37, which was calculated at 84.9% for the 1%/1 mm criteria.

Our results suggest that even for the worst‐case scenarios observed in the cohort, the impact of uncorrected air mapping errors on the dose distribution was small and is unlikely to impact the clinical dose metrics used to generate the adaptive plan. Correction of air volume would add unnecessary time to the already long and involved process of adaptive therapy and will likely introduce new errors. It is also important to note that the oART session currently exceeds 20 min on average at our institution. In this time period, abdominopelvic air cavities may migrate, expand, or disappear, rendering the time‐consuming manual corrections invalid. Our results are in concordance with previous studies that investigated the dosimetric effects of electron density correction for MR‐based oART^29^ and gas overrides in a phantom‐base commissioning of the Ethos workflow.^30^ Both studies agreed that manual correction of air volume is time consuming and has minimal clinical effect in the dose distribution.

Although manual correction of air mapping errors during the oART workflow is not recommended based on our results, algorithm improvements to better handle the air cavity mapping in an automated fashion may still be beneficial, especially for patients that may exhibit even larger daily gas fluctuations than our cohort. Special attention should be taken when air cavities are contained within the CTV such as in rectal cancer. A previous case study on adaptive rectal cancer treatment reported insufficient target coverage due to the intrafraction motion of a large gas pocket and the need for re‐adaptation.^31^ Ultimately, because abdominal gas not only affects accurate dose calculation, but also the image quality of the daily CBCT, air volume near the treatment site should be actively monitored, with proactive implementation steps being taken to reduce intestinal gas while patients are under treatment.

## 5 CONCLUSION

The air mapping accuracy and its dosimetric impact of a recently introduced CBCT‐based oART platform was studied for abdominopelvic oART. Although considerable air mapping inaccuracy was observed, where the daily air volume is usually underestimated in the synthetic CT, the dosimetric impact was found to be small for prostate and nodal treatments. Therefore, online manual correction of air cavities is deemed unnecessary. Because abdominal gas not only affects accurate dose calculation but also the image quality, we recommend active monitoring of air volume near the treatment site and the proactive implementation of strategies to reduce intestinal gas during adaptive radiation treatment.

## AUTHOR CONTRIBUTIONS

Olga M. Dona Lemus designed the project, acquired, and analyzed the data and wrote the manuscript. Sean Tanny contributed to data acquisition and analysis and revised the manuscript. Matthew Webster, Joshua Wancura, Michael Cummings, Hyunuk Jung, Yuwei Zhou, Jihyung Yoon, and Matthew Pacella made significant contribution to the design of the work and revised the manuscript. Dandan Zheng provided mentorship and resources needed for the project, contributed to the conception and design of the work, and revised the manuscript. All authors approved the submitted draft and agreed to be accountable for all aspects of this work.

## CONFLICT OF INTEREST STATEMENT

The authors whose names are listed immediately below certify that they have NO affiliations with or involvement in any organization or entity with any financial interest (such as honoraria; educational grants; participation in speakers’ bureaus; membership, employment, consultancies, stock ownership, or other equity interest; and expert testimony or patent‐licensing arrangements), or non‐financial interest (such as personal or professional relationships, affiliations, knowledge or beliefs) in the subject matter or materials discussed in this manuscript.
